# Prophylactic Activation of Shh Signaling Attenuates TBI-Induced Seizures in Zebrafish by Modulating Glutamate Excitotoxicity through Eaat2a

**DOI:** 10.3390/biomedicines10010032

**Published:** 2021-12-24

**Authors:** James Hentig, Leah J. Campbell, Kaylee Cloghessy, Mijoon Lee, William Boggess, David R. Hyde

**Affiliations:** 1 Department of Biological Sciences, Galvin Life Science Building, University of Notre Dame, Notre Dame, IN 46556, USA; jhentig@nd.edu (J.H.); lcampbe4@nd.edu (L.J.C.); kcloghes@nd.edu (K.C.); 2 Center for Zebrafish Research, Galvin Life Science Building, University of Notre Dame, Notre Dame, IN 46556, USA; 3 Center for Stem Cells and Regenerative Medicine, Galvin Life Science Building, University of Notre Dame, Notre Dame, IN 46556, USA; 4 Department of Chemistry and Biochemistry, University of Notre Dame, Notre Dame, IN 46556, USA; mlee12@nd.edu (M.L.); wboggess@nd.edu (W.B.)

**Keywords:** traumatic brain injury, blunt-force TBI, post-traumatic seizures, zebrafish, sonic hedgehog signaling, purmorphamine

## Abstract

Approximately 2 million individuals experience a traumatic brain injury (TBI) every year in the United States. Secondary injury begins within minutes after TBI, with alterations in cellular function and chemical signaling that contribute to excitotoxicity. Post-traumatic seizures (PTS) are experienced in an increasing number of TBI individuals that also display resistance to traditional anti-seizure medications (ASMs). Sonic hedgehog (Shh) is a signaling pathway that is upregulated following central nervous system damage in zebrafish and aids injury-induced regeneration. Using a modified Marmarou weight drop on adult zebrafish, we examined PTS following TBI and Shh modulation. We found that inhibiting Shh signaling by cyclopamine significantly increased PTS in TBI fish, prolonged the timeframe PTS was observed, and decreased survival across all TBI severities. Shh-inhibited TBI fish failed to respond to traditional ASMs, but were attenuated when treated with CNQX, which blocks ionotropic glutamate receptors. We found that the Smoothened agonist, purmorphamine, increased Eaat2a expression in undamaged brains compared to untreated controls, and purmorphamine treatment reduced glutamate excitotoxicity following TBI. Similarly, purmorphamine reduced PTS, edema, and cognitive deficits in TBI fish, while these pathologies were increased and/or prolonged in cyclopamine-treated TBI fish. However, the increased severity of TBI phenotypes with cyclopamine was reduced by cotreating fish with ceftriaxone, which induces Eaat2a expression. Collectively, these data suggest that Shh signaling induces Eaat2a expression and plays a role in regulating TBI-induced glutamate excitotoxicity and TBI sequelae.

## 1. Introduction

Traumatic brain injuries (TBIs) have tremendous and lasting impacts because they can result in various sequelae and are one of the leading causes of disease burden in the United States [1,2,3]. Post-traumatic seizures (PTS) are a common consequence following TBI [3,4]. Development of PTS is influenced by age at time of injury, type of TBI (blunt vs. penetrating), and injury severity [4,5,6]. Although the incidence rate varies greatly, PTS has been reported as high as 30% in military veterans with TBI, while civilians displayed a 20–50% increased risk of TBI-induced PTS compared to the development of non-acquired epilepsy [7,8]. Furthermore, PTS can result in a cyclic injury pattern in which TBI results in PTS, and during seizure event, another TBI occurs from fall-like events. Several anti-seizure medications (ASMs) exist that rely largely on γ-aminobutyric acid (GABA)- regulated mechanisms to combat uncontrolled excitatory-driven seizures [9]. However, a large portion of individuals that experience TBI-induced PTS are resistant to many ASMs [10,11]. This could be due to TBI resulting in a secondary injury that includes glutamic excitotoxicity [12].

Although rodents are the traditional neurotrauma model due to their recapitulation of a variety of human neurobehavioral deficits following injury, alternative models such as zebrafish have recently emerged [13,14,15,16,17]. Zebrafish possess many features that are advantageous for studying neurotrauma such as an extensively characterized behavioral repertoire [18], recapitulate a variety of human pathologies following a TBI [17], and several orthologs of excitatory amino acid transporters (Eaat), which are thought to play key roles in TBI excitotoxicity and epileptogenesis [19,20]. It was recently demonstrated using a zebrafish *eaat2a* mutant that loss of the glutamate transporter exhibited defects in neuronal function and epileptic seizures originating in the hindbrain of developing zebrafish [21], although it remains unknown if upregulating Eaat2 expression can suppress PTS.

We recently described a scalable blunt-force zebrafish TBI model that produced brain edema, neuroinflammation, cognitive deficits, and PTS, followed by neuronal regeneration [17]. Following injury, components of the Shh signaling pathway were rapidly and highly upregulated, and Shh was found to be important in the injury-induced proliferation. The use of purmorphamine, a Smoothened agonist and Shh signaling activator, was previously shown to ameliorate a variety of disorders, improve behavioral and cognitive outcomes, reduce neuroinflammation, and minimize edema following CNS trauma [22,23]. However, few studies have examined the effects of modulating Shh signaling in relation to PTS and regulating excitotoxicity.

Here, we examined the mechanistic interface between Shh and Eaat2 and demonstrated that prophylactic activation of Shh via purmorphamine attenuated TBI-induced PTS by reducing extracellular glutamate. In contrast, cyclopamine-induced inhibition of the innate Shh response following TBI increased PTS events and prolonged injury-induced edema and cognitive deficits. These data suggest that prophylactic activation of Shh upregulates Eaat2 and combats excitotoxicity, resulting in reduced injury pathologies.

## 2. Materials and Methods

### 2.1. Fish Lines and Maintenance

Adult wild-type *albino*^b4^ zebrafish [24] (Danio rerio) of both sexes were maintained in the Center for Zebrafish Research at the University of Notre Dame Freimann Life Sciences Center. This study used approximately equal numbers of male and female adult zebrafish, 6–18 months old, and 3 to 5 cm in length. All experimental protocols in this study were approved by the University of Notre Dame Animal Care and Use Committee protocol # 18-03-4558.

### 2.2. TBI Induction via Modified Marmarou Weight Drop

Zebrafish were anesthetized in 1:1000 2-phenoxyethanol (2-PE, Sigma-Aldrich, St. Louis, MO, USA) until unresponsive to tail pinch as described in approved IACUC protocol. 2-PE was used because it is approved for anesthesia in zebrafish and has no effect on neuronal function even at higher concentrations [25]. A modified Marmarou weight drop protocol for zebrafish [17,26] was used to administer a blunt-force injury. Following anesthetic, fish were secured onto a clay mold that stabilized the body and exposed the zebrafish head. Undamaged fish were anesthetized, secured to the damage rig, and then returned to an aerated recovery tank. For TBI, fish were anesthetized and either a 1.5 g or 3.3 g ball bearing weight was dropped down a shaft of either 7.6 or 12.7 cm length to produce the desired force. All TBIs were induced between 4:00 and 8:00 p.m., and following the TBI, fish were placed into an aerated tank to recover, and then placed in standard living conditions. At the desired end points, fish were euthanized in 2-PE (1:500 dilution) until either unresponsive to tail pinch or no operculum movement for 30 min, whichever came last as described in an approved IACUC protocol.

### 2.3. Survival and Seizure

Following blunt-force injury and recovery from anesthesia, zebrafish were assessed for post-traumatic seizures within the first 1 h after injury and for 30 min every 12 hpi up to 5 dpi. Fish were observed and scored for displaying the following clonic-seizure metrics defined in the zebrafish behavior catalog [18]: ataxia (ZBC 1.9), bending (ZBC 1.16), circling (ZBC 1.32), and corkscrew swimming (ZBC 1.37). To assess survival and latent seizure activity, untreated and cyclopamine/purmorphamine-treated fish were observed for 30 min every 12 hpi and observed for the same metrics: mortality was determined as no operculum movement over the 30 min observation period, and latent post-traumatic seizures as defined by the same ZBC metrics. Shh-modulated survival and seizure activity was calculated as an average of each group (*n* = 100 fish per control/experimental group).

### 2.4. Fluid Content Measurement

Edema was measured using a modified Hoshi protocol [26,27]. Whole brains (undamaged, sTBI, or sTBI treated fish *n* = 9 fish control/experimental group) were isolated from control and injured fish at 1 or 3 dpi, weighed, dried at 60 °C for 12 h, and weighed again, with the percent fluid calculated using the following formula:

$$\%\mathrm{Fluid}=\frac{\mathrm{wet}\;\mathrm{weight}-\mathrm{dry}\;\mathrm{weight}}{\mathrm{wet}\;\mathrm{weight}}\times 100\%$$

### 2.5. Learning

Learning was assessed as previously described [28]. Treated and untreated naïve undamaged control or blunt-force-damaged fish were individually placed into the shuttle box and examined at 1, 3, 5, 7, or 14 dpi, and assessed for learning with no fish being tested twice. A positive trial was recorded when the fish swam to the other stall in response to the red-light visual stimulus However, a failed trial was recorded if the fish failed to swim to the other stall within the 15 s of the red-light exposure, followed by a simultaneous pulsating electric current (20 V, 1 mA) for up to 15 s (10 electrical shocks/15 s). This was repeated until the fish successfully completed 5 consecutive trials. The number of trials that each fish required to learn were determined for each treatment group and averaged for each experiment, (*n* = 9 fish control/experimental group). A two-way ANOVA and Tukey’s post hoc test were performed to statistically compare the undamaged control fish and the different damage groups.

### 2.6. Memory

Memory was assessed as previously described [28]. Purmorphamine-treated and untreated naïve undamaged fish were individually placed into the shuttle box testing apparatus. Fish were acclimated in a dark and quiet room for 15 min. A red-light visual and a pulsating electric current (20 V, 1 mA) were simultaneously applied until the fish swam halfway across the testing tank. Presentation of the visual stimulus and electrical current was repeated 25 times (training) with 30 s of rest between intervals. Fish were then tested 15 min later by exposing the fish only to the red-light stimulus (initial testing period) 25 times. The number of successful trials, in which the fish did not require the electric shock to cross the tank, was counted to generate the initial testing baseline. Once all fish were tested, they were randomly selected for either the undamaged control group or were administered a severe blunt-force injury. To assess immediate recall, undamaged and experimental groups were then subjected to a post-sham/injury testing period consisting of 25 iterations 4 h/hpi. To test delayed recall, four days after the initial training and testing period, fish were randomly selected and given a severe blunt-force injury and allowed to recover for 4 h/hpi. Both undamaged and experimental groups were then subjected to a post-sham/injury testing period of 25 iterations. For both recall examinations, the percent difference in the number of successful post-sham/injury trials relative to the number of initial successful trials was calculated and averaged for each group.

### 2.7. Pharmacological Agents

**Figure 1:**
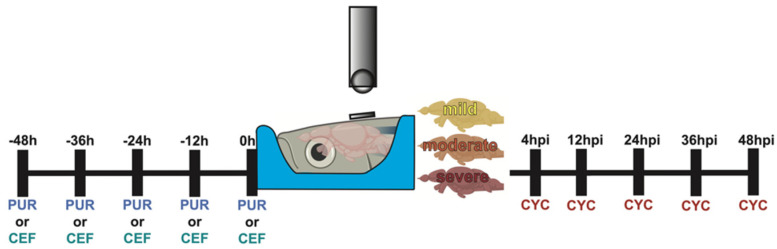
Experimental timeline of Shh modulation. Graphical representation of the dosing schedule of purmorphamine, ceftriaxone, and cyclopamine in relation to the timing of the TBI.

Sonic hedgehog and EAAT2 modulation was performed as illustrated in Figure 1.

Purmorphamine: Undamaged fish were intraperitoneally (IP) injected with ~40 µL of 10 µM purmorphamine (Sigma-Aldrich, St. Louis, MO, USA) using a 30 gauge needle every 12 h for 48 h (0, 12, 24, 36, and 48 h).

Cyclopamine: Fish were exposed to sTBI and IP injected with ~40 µL of 2 mM cyclopamine (Sigma-Aldrich, St. Louis, MO, USA) using a 30 gauge needle at 4, 12, 24, 36, and 48 hpi.

Ceftriaxone: Undamaged fish were IP injected with ~40 µL of 10 mM ceftriaxone (Sigma-Aldrich, St. Louis, MO, USA) using a 30 gauge needle ever 12 h for 48 h (0, 12, 24, 36, and 48 h).

6-Cyano-7-nitroquinoxaline-2,3-dione (CNQX): Fish were exposed to sTBI and IP injected with ~40 µL of 2 mM CNQX (Sigma-Aldrich, St. Louis, MO, USA) and 2 mM cyclopamine using a 30 gauge needle at 4, 12, 24, 36, and 48 hpi.

Valproic acid (VPA): Fish were exposed to sTBI and IP injected with ~40 µL of 2 mM VPA (Sigma-Aldrich, St. Louis, MO, USA) and 2 mM cyclopamine using a 30 gauge needle at 4, 12, 24, 36, and 48 hpi.

Gabapentin (GABAP): Fish were exposed to sTBI and IP injected with ~40 µL of 3 mM GABAP (Sigma-Aldrich, St. Louis, MO, USA) and 2 mM cyclopamine using a 30 gauge needle at 4, 12, 24, 36, and 48 hpi.

### 2.8. Glutamate Excitotoxicity

Undamaged fish treated with either purmorphamine, cyclopamine, ceftriaxone, or a combination of the three were placed in a tank containing 5 mM glutamate (Sigma-Aldrich, St. Louis, MO, USA) in the tank water for 30 min. The time of the first seizure event per fish was recorded (once a fish experienced a seizure event, they were removed and placed into a second tank with 5 mM glutamate for the remainder of the 30 min as to avoid counting a second seizure as another unique event), and the percent of fish in each cohort that experienced at least one seizure was quantified. Following the 30 min glutamate exposure, all surviving fish were transferred to a tank containing only standard tank water and assessed for an additional 30 min for survival, and the percent of fish in each cohort that survived was quantified (*n* = 90 fish control/experimental group). Statistical analysis was performed with a one-way ANOVA followed by Tukey’s post hoc test.

### 2.9. Quantitative Real-Time PCR (qRT-PCR)

Total RNA was isolated and purified from whole telencephalons and cerebellums from ten adult untreated and purmorphamine-treated undamaged fish using Trizol extraction. The RNA was converted to cDNA from 1 ug of RNA using qScript cDNA SuperMix (VWR International, Radnor, PA, USA) as previously described [29]. Taqman probes were used according to the manufacturer’s instructions with 10 ng of cDNA. Taqman probes (Thermo Fisher) *gli1* (Dr03093665_m1), *eaat1a* (Dr03120588_m1), *eaat2a* (Dr03119707_m1), *eaat3* (Dr03108401_m1), and *il1β* (Dr03114367_g1) were used for quantitative real-time PCR (qRT-PCR) and the data were normalized to 18 s rRNA (Hs03003631_g1) in each well. Data were acquired using the ABI StepOnePlus Real-Time PCR System (Applied Biosystems). Cycling conditions were as follows: 2 min at 50 °C, 10 min at 95 °C, and 40 cycles of 15 s at 95 °C and 1 min at 60 °C with data collection occurring following the 60 °C extension step. The ΔΔCT values were calculated and used to determine the log2-fold changes [30] of *gli1*, *eaat1a*, *eaat2a*, and *eaat3*. Expression levels were examined in technical and biological triplicate.

### 2.10. Microdialysis and Glutamate Quantification

Post-injury extracellular glutamate concentrations were determined using a modified Puppala protocol [31]. Undamaged, sTBI, and sTBI fish treated with either purmorphamine, cyclopamine, or ceftriaxone were collected at 30 min post-injury, 12 hpi, 36 hpi, or 5 dpi and were euthanized with 1:500 2-PE. Each fish was immobilized by modeling clay, and the skull removed. A microdialysis probe (CMA7, 2 mm membrane length; CMA/Microdialysis, N. Chelmsford, MA, USA), which was rinsed and equilibrated for 30 min, was slowly inserted under a dissection microscope into the cerebellum along the rostral-caudal axis. The corpus cerebelli was dialyzed with sterile Ringer’s solution (147 mM Na^+^, 2.3 mM Ca^2+^, 4 mM K^+^, and 155.6 mM Cl^−^; pH = 6.0) at a flow rate of 5 μL/min. Dialysate fractions were collected for 30 min and immediately stored at −20 °C. Following the manufacturer’s instructions (Glutamate-Glo™ Assay, Promega, Madison, WI, USA), samples were thawed and 50 µL was incubated for 1 h with 50 µL mastermix of Luciferin detection solution, reductase, reductase substrate, glutamate dehydrogenase, and NAD. Samples were analyzed on a SpectraMax M5 plate reader (Molecular Devices, San Jose, CA, USA), and sample RLU values were compared to a glutamate serial dilution standard curve to quantify glutamate concentration. Comparisons were made using a one-way ANOVA followed by either Tukey’s or Dunnett’s multiple comparison post hoc test.

### 2.11. Immunoblot

Total protein collection and immunoblotting techniques were performed as previously described [32], with minor modifications. Five cerebellums were pooled for each experimental replicate. Both chicken anti-Eaat2a (1:1000, gift of Neuhauss lab) [33] and rabbit anti-Gapdh (1:2500; cat #: ab210113, Abcam, Cambridge, UK) polyclonal anti-sera were incubated on membranes (0.45 µm PVDF, VWR, Radnor, PA, USA) that were blocked overnight at 4 °C in 1 × TBS/5% nonfat dry milk/0.1% Tween 20. Membranes were washed 4 × 15 min in 1 × TBS/0.1% Tween 20. The membranes were incubated with anti-chicken or anti-rabbit peroxidase secondary antibody (1:10,000, chicken, cat #: A9046, Sigma-Aldrich, St. Louis, MO, USA; rabbit, NA934, VWR International, Radnor, PA, USA), washed, and detected as described previously [32]. The blot was incubated with the ECL-Prime detection system (Fischer Scientific, Waltham, MA, USA) and exposed to X-ray film.

### 2.12. Statistical Analysis

With regard to all data within this study, except for microdialysis and immunoblot, each experiment was obtained from three independent trials of at least 3 fish per trial. The data are expressed as the mean ± SE of the mean. Data sets were analyzed in Prism 8 (GraphPad) with either Student’s *t*-test for single pairwise comparisons with control, one-way or two-way ANOVA followed by either Tukey’s or Dunnett’s post hoc test for multiple comparisons, or the Friedman test for repeated-measures data. The statistical test used is stated in each figure legend.

## 3. Results

### 3.1. Shh Inhibition Increases TBI-Induced Post-Traumatic Seizures

**Figure 2:**
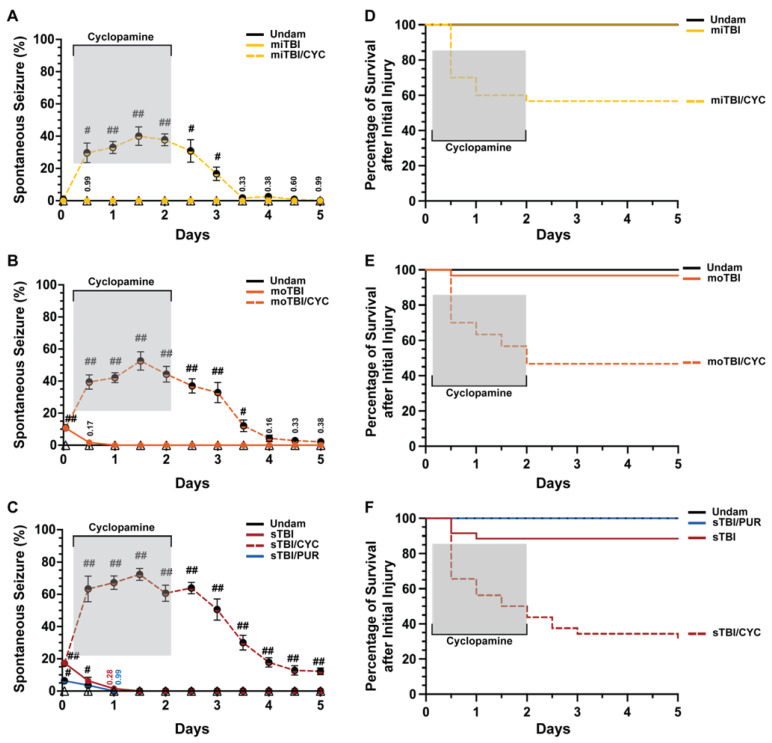
Modulation of Shh alters frequency of TBI-induced PTS events. (**A**–**C**) Percentage of fish that displayed spontaneous seizure events spanning the timeframe from within 1 hpi to 5 dpi with and without Shh modulation. (**D**–**F**) Percentage of survival across 5 days of fish who initially survived the primary injury across mi-, mo-, and sTBI with and without Shh modulation. Statistical analyses of the repeated-measures data were performed with the Friedman test, *n* = 100 fish per control/experimental group, grey box denotes period of cyclopamine administration, # *p* < 0.05, ## *p* < 0.01.

Shh was identified as a key pathway that is activated following damage to the central nervous system (CNS) in the adult zebrafish and is required for injury-induced regeneration [17,34,35]. It was shown that following TBI in zebrafish and rodents, genes encoding Shh signaling components were upregulated as early as 6 h post-injury (hpi) [17,36]. Additionally, Shh activation attenuated sequelae following various forms of CNS trauma [23,37,38,39]. We examined undamaged and TBI fish that were either untreated or treated with the Smo antagonist cyclopamine (a Shh signaling inhibitor) at 4, 12, 24, 36, and 48 hpi. Undamaged fish, whether untreated or cyclopamine-treated, displayed no seizure behavior, while untreated TBI fish displayed modest, but significant, PTS in a severity-dependent manner (Figure 2A–C). No PTS was observed within 1 hpi following a mild TBI (miTBI, Figure 2A), while moderate TBI (moTBI) induced PTS in a significant percentage of fish immediately following injury (10.66% ± 1.37%, Figure 2B) and this was further elevated following severe TBI (sTBI, 17.44% ± 1.54%, Figure 2C). Untreated TBI fish exhibited a rapid decrease in seizure behavior, with no clonic-tonic seizures observed in moTBI by 1 day post-injury (dpi) and in sTBI by 1.5 dpi (Figure 2B,C). In contrast, cyclopamine-treated TBI fish (TBI/CYC) displayed a significantly increased percentage of fish with PTS compared to their matched counterparts. A significantly increased percentage of miTBI/CYC fish experienced PTS at 12 hpi (29.66% ± 6.10%, *p* < 0.01) and the percentage peaked at 1.5 dpi (40% ± 5.76%, *p* < 0.01, Figure 2A). Similarly, and in a severity-dependent manner, increased percentages of moTBI/CYC (39.4% ± 4.43%, *p* < 0.01) and sTBI/CYC (63.33% ± 7.99%, *p* < 0.01) fish were observed at 12 hpi and peaked at 1.5 dpi (moTBI/CYC: 52.57% ± 5.70%, *p* < 0.01, sTBI/CYC: 72.33% 3.73%, *p* < 0.01, Figure 2B,C). Although cyclopamine treatment ceased at 2 dpi, all severities continued to experience heightened seizure activity for a period of time. The percentage of fish exhibiting PTS noticeably decreased in miTBI and moTBI by 3.5 and 4 dpi, respectively (Figure 2A,B). However, sTBI/CYC fish continued to experience significant seizure activity out to 5 dpi (*p* < 0.01, Figure 2C).

We also observed stark differences in survival between TBI fish and cyclopamine-treated TBI fish (Figure 2D–F). Over 5 days following injury, all miTBI fish survived (Figure 2D), and although moTBI and sTBI experienced some mortality, they largely survived (moTBI: 96.66%, sTBI: 85.71%, Figure 2E,F). In contrast, only 56.66% of miTBI/CYC fish survived 5 dpi (Figure 2D), while survival continued to decrease in both moTBI/CYC (46.66%, Figure 2E) and sTBI/CYC fish (28.12%, Figure 2F). For all severities, the greatest mortality occurred at 12 hpi, shortly after initiating Shh pathway inhibition. For miTBI/CYC and moTBI/CYC, all death occurred during cyclopamine treatment with no mortality following the cessation of cyclopamine. In contrast, sTBI/CYC fish continued to experience mortality following the end of cyclopamine treatment, although slowed, out to 5 dpi (Figure 2F). Collectively, these data suggest that inhibiting Shh signaling plays a role in seizure activity and overall survival following TBI.

### 3.2. Blocking Ionotropic Glu Receptors Inhibits TBI-Induced PTS

**Figure 3:**
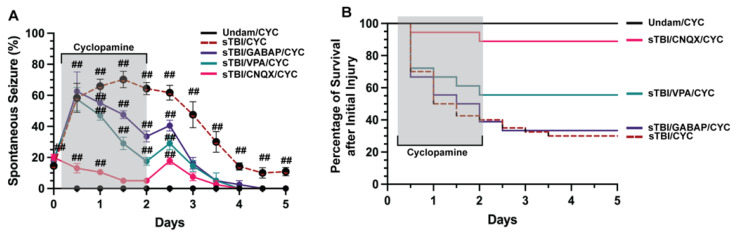
CNQX attenuates PTS in Shh-inhibited fish. (**A**) Percentage of undamaged and sTBI that were Shh inhibited (cyclopamine-treated) and cotreated with either valproic acid (VPA), gabapentin (GABAP), or CNQX that displayed spontaneous seizure events across 5 dpi. (**B**) Quantification of survival across 5 days of undamaged or sTBI Shh-inhibited fish cotreated with VPA, GABAP, or CNQX. Statistical analysis of the repeated-measures data was performed with the Friedman test, *n* = 100 fish per control/experimental group, grey box denotes period of cyclopamine/(VPA or GABAP or CNQX) administration, ## *p* < 0.01.

Neuronal signaling is finely tuned and small deviations that go uncontrolled can manifest in various aberrant neurobehaviors, including seizures [40,41]. Developmental and acquired events can induce seizures, usually resulting from misregulation of neurotransmitters glutamate or GABA [42,43]. We examined if TBI-induced seizures that were exacerbated following inhibition of Shh signaling were predominately glutamate or GABA driven. We coinjected sTBI fish that were treated with cyclopamine at 4, 12, 24, 36, 48 hpi with either the ionotropic glutamate receptor antagonist 6-cyano-7-nitroquinoxaline-2,3-dione (CNQX), valproic acid (VPA) a common first-line ASM [44], or the synthetic GABA analog gabapentin (GABAP). Undamaged fish treated with cyclopamine displayed no seizures, while sTBI/CYC fish experienced rapid and significantly increased percentage of fish with PTS seizure at 12 hpi (58.33% ± 9.36%, *p* < 0.01, Figure 3A), peak percentage at 1.5 dpi (70.16% ± 5.29%, *p* < 0.01) and remained significantly elevated after ceasing cyclopamine (5 dpi: 10.83% ± 2.71%, *p* < 0.01, Figure 3A). Cyclopamine-treated sTBI that were coinjected with either VPA or GABAP (VPA/CYC and GABAP/CYC, respectively) showed similar percentages of PTS-experiencing sTBI fish that were only treated with cyclopamine. At 12 hpi, both VPA/CYC- and GABAP/CYC-treated fish experienced significantly increased percentages of fish with PTS (VPA/CYC: 57.5% ± 3.35%, *p* < 0.01, GABAP/CYC: 62.5% ± 5.59%, *p* < 0.01, Figure 3A). In contrast, the percentage of sTBI cyclopamine-treated fish that were coinjected with CNQX (CNQX/CYC) with PTS was significantly decreased at 12 hpi compared to sTBI/CYC fish (*p* < 0.05), although still increased compared to undamaged cyclopamine-injected fish (13% ± 1.34%, *p* < 0.01, Figure 3A). By 1 dpi, VPA and GABAP treatments resulted in a noticeably decreased percentage of PTS fish, while CNQX treatment significantly reduced the percentage of PTS fish greater than either VPA or GABAP treatment (CNQX/CYC vs. VPA/CYC, *p* < 0.01, CNQX/CYC vs. GABAP/CYC, *p* < 0.01, VPA/CYC vs. GABAP/CYC, *p* = 0.26, Figure 3A). Additionally, CNQX reduced the percentage of PTS to a level similar to Undam/CYC by 1.5 dpi compared to 3 dpi for VPA/CYC- and GABAP/CYC-treated fish (Figure 3A). Similarly, cyclopamine treatment (sTBI/CYC) negatively impacted survival (5 dpi: 27.5%), as did sTBI/CYC fish cotreated with GABAP (5 dpi: 33.33%, Figure 3B). sTBI fish cotreated with cyclopamine and VPA displayed a slight improvement in survival (55.6%). In contrast, sTBI/CYC fish that were cotreated with CNQX experienced dramatically improved survival (88.89%, Figure 3B), to a level that was similar to undamaged fish. Collectively, these data suggest that traditional GABA-driven ASMs had minimal influence on TBI-induced PTS and survival, while blocking glutamate receptors with CNQX attenuated PTS and improved survival, suggesting that TBI-induced PTS may be due to glutamic excitotoxicity.

### 3.3. Shh Activation Combats Excitotoxicity and Upregulates Eaat2a

**Figure 4:**
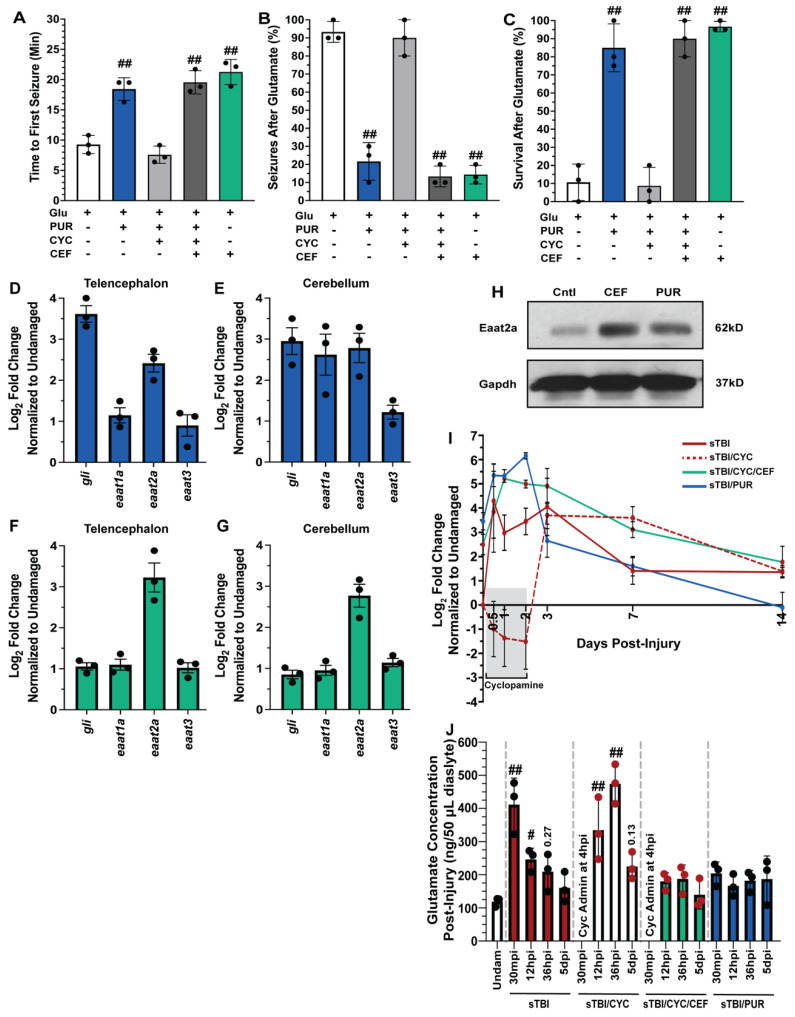
Shh activation combats excitotoxicity and upregulates Eaat2a. Undamaged fish with and without Shh modulation and ceftriaxone treatment were exposed to 5 mM glutamate in fish water (*n* = 90 fish per control/experimental group). (**A**) Quantification of time to first seizure, (**B**) percent of the group to display at least 1 seizure event, and (**C**) percent of the group that survived for 1 h. (**D**,**E**) Expression of Shh component, *gli,* and excitatory amino acid transporter (*eaat*) genes by qRT-PCR revealed that purmorphamine increased *gli* and *eaat2a* mRNAs in both undamaged telencephalons and cerebellums (*n* = 3 per control/experimental group, with 5 pooled telencephalons or cerebellums/trial). (**F**,**G**) Administration of ceftriaxone increased *eaat2a* mRNA expression levels without Shh activation in undamaged telencephalons and cerebellums (*n* = 3 per control/experimental group, with 5 pooled telencephalons or cerebellums/trial). (**H**) Expression of Eaat2a in the undamaged cerebellum (5 pooled cerebellums per control/experimental group) following either purmorphamine or ceftriaxone treatment was assessed by immunoblot, using GAPDH as a loading control (H, top and lower bands, respectively). Eaat2a protein expression was increased following either purmorphamine or ceftriaxone treatment compared to controls. (**I**) Expression of *eaat2a* by qRT-PCR in sTBI fish with either Shh signaling activated, inhibited, or inhibited and cotreated with ceftriaxone at 12 hpi to 14 dpi (*n* = 3 per control/experimental group, with 5 pooled cerebellums/group). (**J**) Microdialysis was performed to quantify extracellular glutamate levels in undamaged and sTBI fish with and without Shh modulation and ceftriaxone treatment at 30 min post-injury, 12 and 36 hpi, and 5 dpi (*n* = 3 per control/experimental group). In panels A-C, the plus sign (+) denotes application of the reagent and the minus sign (-) denotes the absence of the reagent. Grey box denotes period of cyclopamine administration. Statistical analyses were performed with a one-way ANOVA followed by Dunnett’s multiple comparison post hoc test, # *p* < 0.05, ## *p* < 0.01.

TBIs result in a wide breadth of pathologies, with excitotoxicity contributing to many TBI-induced sequelae [43]. Previously, Shh signaling was reported to reduce excitotoxicity following middle cerebral artery occlusion in rodents [45,46], though it was never examined following TBI in adult zebrafish. To test if Shh signaling could affect glutamate-induced excitotoxicity, undamaged fish were either left untreated, treated with the Smo agonist purmorphamine, which is a Shh signaling activator, or treated with both purmorphamine and cyclopamine. Fish were then challenged with 5 mM glutamate in their tank water for 30 min and the time of the first seizure, percentage of the cohort to seize, and the overall survival of the cohort was quantified. Untreated fish experienced rapid seizure onset with the first seizure recorded an average of 9.26 min (±0.86 min), the majority of the fish (93.3% ± 3.3%) experienced a seizure, and survival was low (10.6% ± 5.8%, Figure 4A–C). In contrast, purmorphamine-treated fish significantly warded off seizure activity, with the average first recorded seizure event at 18.43 min (±1.07 min, *p* < 0.01), significantly experienced fewer fish with a seizure during the glutamate challenge (21.6% ± 6.0%, *p* < 0.01), and survival was significantly improved (85% ± 7.63%, *p* < 0.01, Figure 4A–C). The attenuation observed in purmorphamine-treated fish was lost when co-treated with cyclopamine, as time of first seizure was reduced to 7.57 min (±0.82 min, *p* = 0.61), 90% (±5.77%, *p* = 0.95) of the fish displayed seizure behavior, and the cohort experienced low survival (8.66% ± 5.92%, *p* = 0.99, Figure 4A–C).

The findings from the glutamate challenge suggested that Shh signaling activation may upregulate one or more excitatory amino acid transporters (Eaat), which would reduce excessive extracellular glutamate following TBI. Both humans and zebrafish have multiple Eaat, though Eaat1 (Glast) and Eaat2 (Glt-1) are the most prominent. To identify which, if any, Eaat may be influenced by Shh activation, we performed qRT-PCR using RNA collected from telencephalons and cerebellums of untreated and purmorphamine-treated undamaged fish. As an internal control, we confirmed Shh activation by observing increased *gli1* expression (downstream targe of Shh signaling) in undamaged purmorphamine-treated brains compared to untreated brains (Figure 4D,E). We then examined *eaat1a*, *eaat2a*, and *eaat3* expression (paralogs *eaat1b* and *eaat2b* are expressed at extremely low levels in the zebrafish brain) [47] and observed increased expression of only *eaat2a* in both the telencephalon and the cerebellum of purmorphamine-treated undamaged brains (Figure 4D,E).

Ceftriaxone was reported to upregulate Eaat2 expression in rodents and zebrafish [48,49]. We treated undamaged fish with 10 mM ceftriaxone every 12 h for 60 h and then collected telencephalons and cerebellums for qRT-PCR analysis. We did not see upregulation of *gli1*, *eaat1a*, *or eaat3*, but did observe increased *eaat2a* expression following ceftriaxone treatment in both the telencephalon and the cerebellum (Figure 4F,G). We also observed increased protein expression of Eaat2a in both purmorphamine and ceftriaxone-treated undamaged brains relative to untreated controls (Figure 4H). In the glutamate challenge, the severe negative effects observed in the purmorphamine/cyclopamine double-treated fish were suppressed by the ceftriaxone treatment and upregulating *eaat2a* expression. Both triple-treated (purmorphamine/cyclopamine/ceftriaxone) and ceftriaxone-only-treated fish exhibited delayed seizure onset with average time of first seizures observed at 19.54 min (±1.11 min) and 21.25 min (±1.2 min), respectively (Figure 4A). Both the triple-treated and ceftriaxone-only-treated groups experienced significantly lower percentage of seizures compared to untreated fish (triple treated: 13.3% ± 3.3%, *p* < 0.01, ceftriaxone only: 14.33% ± 2.96%, *p* < 0.01, Figure 4B), and both groups exhibited significantly improved survival (triple treated: 90% ± 5.77%, *p* < 0.01, ceftriaxone only: 96.6% ± 1.6%, *p* < 0.01, Figure 4C).

We next examined the temporal expression of *eaat2a* relative to injury. We performed qRT-PCR of *eaat2a* in sTBI fish at 12 hpi, 1, 2, 3, 7, and 14 dpi with Shh modulation (Figure 4I). Following injury, untreated sTBI fish exhibited upregulated *eaat2a* expression at 12 hpi, which then gradually decreased to near undamaged levels by 7 and 14 dpi (Figure 4I). In sTBI/CYC fish the initial expression was noticeably repressed during cyclopamine treatment through 2 dpi, followed by a robust increase in *eaat2a* expression between 3 and 7 dpi that returned near basal levels at 14 dpi (Figure 4I). In contrast, fish that were treated with both ceftriaxone and cyclopamine exhibited a rapid increase in *eaat2a* expression that peaked at 2 dpi, before decreasing through 14 dpi, though it was still elevated relative to untreated fish (Figure 4I). Similarly, sTBI/PUR fish displayed elevated *eaat2a* expression from 12 hpi through 2 dpi, before declining and reaching untreated undamaged levels by 14 dpi (Figure 4I).

We next examined the effect of manipulating Shh activation on TBI-induced glutamate excitotoxicity. Due to the heightened and peak seizure activity observed following cyclopamine treatment at 1.5 days (36 hpi), we examined the concentration of extracellular glutamate at multiple time-points (30 min, 12 h, 36 h, and 5 days post-injury) from sTBI or sTBI fish treated with either cyclopamine, ceftriaxone/cyclopamine, or purmorphamine, and compared to uninjured controls. Glutamate levels in sTBI fish 30 min post-injury (mpi) and 12 hpi were significantly higher compared to undamaged fish (sTBI 30 mpi: 411.66 ng ± 46.36 ng, *p* < 0.01, sTBI 12 hpi: 246.3 ng ± 19.53 ng, *p* < 0.05, Undam: 118.66 ng ± 7.83 ng, Figure 4J), and although still elevated, decreased in sTBI fish at 36 hpi (sTBI 36 hpi: 209 ng ± 32.57, *p* = 0.27, Figure 4J). First cyclopamine administration occurred at 4 hpi as it has been shown that injury-induced upregulation of Shh signaling components occurred as early as 6 hpi [17]. Therefore, assessment of glutamate levels in cyclopamine-treated fish at 30 mpi was not possible. Cyclopamine-treated sTBI fish at 12 hpi possessed significantly elevated glutamate levels compared to undamaged fish (sTBI/CYC 12 hpi: 334.6 ng ± 54.29 ng, *p* < 0.01), which was further elevated at 36 hpi (sTBI/CYC 36 hpi: 474.3 ng ± 34.36 ng) compared to both undamaged (*p* < 0.01) and sTBI 36 hpi fish (*p* < 0.01, Figure 4J). Cyclopamine treatment ended at 60 hpi, and while glutamate levels were still elevated at 5 dpi, they were statistically not different than undamaged levels (sTBI/CYC 5 dpi: 225 ng ± 23.47 ng, *p* = 0.13, Figure 4J). The sTBI/CYC/CEF fish exhibited glutamate levels following injury that were not statistically different from undamaged fish at any timepoint (Figure 4J). A similar effect was seen in purmorphamine-treated fish, where extracellular TBI-induced glutamate levels were slightly elevated, but not statistically different, relative to undamaged fish at any time point (Figure 4J). Collectively, these data suggest that purmorphamine-mediated Shh attenuates excitotoxicity by regulating extracellular glutamate via Eaat2a.

### 3.4. Shh Activation Reduces Edema, Neuroinflammation, and PTS following TBI

**Figure 5:**
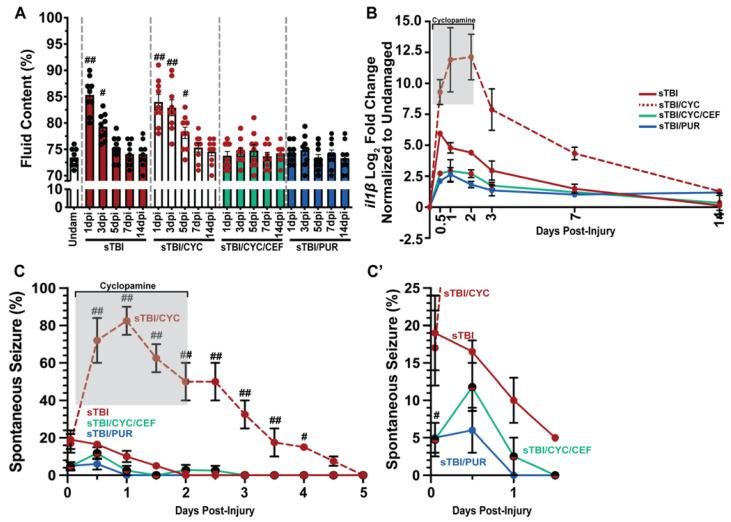
Prophylactic Shh activation attenuates TBI-induced edema, neuroinflammation, and PTS. (**A**) Histogram quantifying brain edema in undamaged and sTBI fish with and without Shh modulation and ceftriaxone treatment across 1 to 14 dpi (*n* = 9 fish per control/experimental group). (**B**) Expression of *il1β* by qRT-PCR in the cerebellum of sTBI fish with and without Shh modulation and ceftriaxone treatment compared undamaged fish (*n* = 3 per control/experimental group, with 5 pooled cerebellums/trial). (**C**) Percentage of fish that displayed spontaneous seizure events spanning the timeframe of from within 1 hpi to 5 dpi with and without Shh modulation and ceftriaxone treatment (*n* = 100 fish per control/experimental group). (**C’**) Expanded view of early post-traumatic seizure percentage. Grey box denotes period of cyclopamine administration. Statistical analyses were performed with either a one-way ANOVA followed by Tukey’s multiple comparison post hoc test (**A**), a two-way ANOVA followed by Dunnett’s multiple comparison post hoc test (**B**), or the Friedman test of the repeated-measures data (**C**), # *p* < 0.05, ## *p* < 0.01.

Increased brain edema and neuroinflammation are highly correlated with increased PTS following TBI [50], while glutamic excitotoxicity increases edema and neuroinflammation [51]. Based on our data, we hypothesized that reducing glutamic excitotoxicity and extracellular glutamate levels post-TBI would also decrease brain edema. We quantified edema, measured as percentage of brain fluid, in undamaged and sTBI fish at 1, 3, 5, 7, and 14 dpi with and without Shh modulation. Following injury, sTBI fish displayed significantly increased brain edema at 1 and 3 dpi compared to undamaged brains (undam: 73.4% ± 0.55%, sTBI 1 dpi: 85.3% ± 1.13%, *p* < 0.01, 3 dpi: 79.3% ± 0.84%, *p* < 0.05, Figure 5A). By 5 dpi, brain edema in sTBI fish is reduced near undamaged levels and remains through 14 dpi. In contrast, sTBI with cyclopamine displayed prolonged and significantly elevated edema 1–5 dpi compared to undamaged brains (sTBI/CYC 1 dpi: 84% ± 1.43%, *p* < 0.01, 3 dpi: 82.88% ± 1.51%, *p* < 0.01, 5 dpi: 78.1% ± 1.08%, *p* < 0.05) before decreasing to undamaged levels by 7 dpi (Figure 5A). However, when ceftriaxone is administered to upregulate *eaat2a* expression in sTBI/CYC fish no significant brain edema was observed at any time point (Figure 5A). Similarly, when sTBI fish were treated with purmorphamine (sTBI/PUR), we again observed no significant brain edema following TBI at any time point (Figure 5A).

In relation to edema, Shh was also reported to affect neuroinflammation through the regulation il1β expression [39,52], which is often used as a critical biomarker in human TBI [53] and known to have a role in zebrafish injury-induced regeneration [54]. We performed qRT-PCR on cerebellar RNA following damage and Shh modulation to assess the expression of il1β. sTBI fish displayed moderate increase in il1β that gradually declined returning near undamaged levels of expression by 7 and 14 dpi (Figure 5B). In contrast, sTBI fish treated with cyclopamine displayed greater expression of il1β, which persisted through the entire cyclopamine treatment followed by a decrease in expression, though it was still elevated at 14 dpi (Figure 5B). However, when sTBI fish were treated with cyclopamine and ceftriaxone, a large reduction in il1β expression was observed relative to sTBI and sTBI/CYC fish (Figure 5B). Similarly, purmorphamine treatment exhibited a reduced il1β expression similar to sTBI/CYC/CEF fish (Figure 5B).

The influence of Shh modulation on PTS (Shh activation decreasing PTS, and inhibition increasing PTS, Figure 2C), and glutamic excitotoxicity suggested that PTS might be suppressed by CEF-mediated increase in *eaat2a* expression. As observed previously (Figure 2), cyclopamine-treated sTBI fish displayed increased seizure activity that persisted significantly to 4 dpi compared to untreated sTBI fish (sTBI/CYC: 15% ± 2.48%, *p* < 0.05, Figure 5C). In contrast, when sTBI fish were treated with cyclopamine and ceftriaxone to upregulate *eaat2a*, the percentage of fish exhibiting PTS was significantly reduced (sTBI/CEF/CYC: 4.75% ± 0.92%, *p* < 0.05, Figure 5C,C’). Similarly, purmorphamine-treated sTBI fish displayed significantly reduced percentage of PTS fish compared to untreated sTBI fish (sTBI/PUR: 5% ± 0.81%, sTBI: 19% ± 2.08%, *p* < 0.05), with no seizure behavior observed in purmorphamine-treated fish after 12 hpi (Figure 5C,C’). Collectively, these data suggest that the attenuation seen in purmorphamine-treated fish may be due to the reduction in brain edema, neuroinflammation, and glutamate excitotoxicity, areas known to be influenced by Shh modulation.

### 3.5. Prophylactic Shh Activation Attenuates TBI-Induced Cognitive Deficits

**Figure 6:**
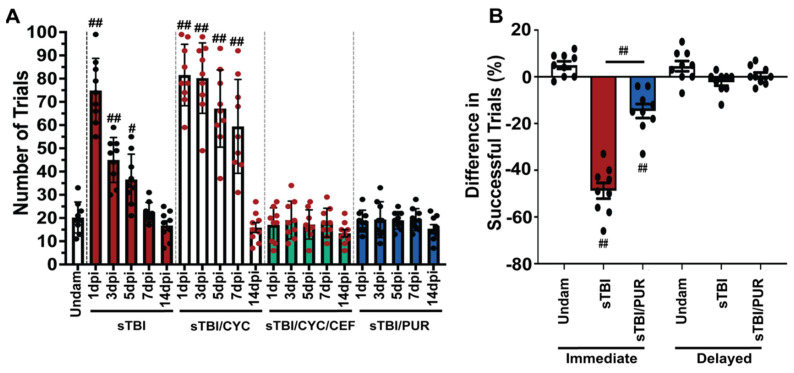
Cognitive impairments are attenuated by prophylactic Shh activation. (**A**) Histogram quantifying the number of trials required for associative learning assay of undamaged and either untreated or Shh-modulated sTBI fish at 1, 3, 5, 7 and 14 dpi (*n* = 9 fish per control/experimental group). (**B**) Quantification of immediate and delayed recall of undamaged, sTBI, and sTBI/PUR fish (*n* = 9 fish per control/experimental group). Statistical analyses were performed with a one-way ANOVA followed by Tukey’s multiple comparison post hoc test, # *p* < 0.05, ## *p* < 0.01.

Purmorphamine treatment has also been shown to improve the neurobehavioral and cognitive recovery following CNS trauma [23,37,38,39]. Zebrafish was previously shown to have a significant learning and memory impairment following sTBI, which rapidly recovered in 4–7 dpi [17,28]. Therefore, we examined the potential of purmorphamine preconditioning to impact TBI-induced cognitive deficits in zebrafish. We assessed associative learning with the shuttle-box assay [17,28], which uses a visual stimulus and an electrical shock as a negative behavioral reinforcement. Undamaged control fish required an average of 20 ± 2.22 trials (Figure 6A) to master the assay (completing 5 consecutive positive trials without the negative reinforcement). Untreated sTBI fish exhibited significant deficits at 1, 3, and 5 dpi (sTBI 1 dpi: 75 ± 4.6 trials, *p* < 0.01, 3 dpi: 45 ± 3.22 trials, *p* < 0.01, 5 dpi: 36 ± 3.6 trials, *p* < 0.05) relative to undamaged control fish, which gradually decreased over time to return statistically to undamaged levels at 7 dpi (22 ± 1.38 trials, Figure 6A). Significant cognitive deficits persisted in Shh-inhibited fish out to 7 dpi relative to undamaged fish (sTBI/CYC 1 dpi: 82 ± trials, *p* < 0.01, 3 dpi: 80 ± trials, *p* < 0.01, 5 dpi: 67 ± trials, *p* < 0.01, 7 dpi: 59 ± trials, *p* < 0.01) before returning to near undamaged levels at 14 dpi (sTBI/CYC 14 dpi: 16 ± trials, *p =* 0.98). Additionally, the sTBI/CYC fish exhibited significant deficits at 3–7 dpi compared to untreated sTBI fish (*p* < 0.01, Figure 6A). However, these deficits in the sTBI/CYC fish were rescued by co-treating fish with ceftriaxone, which did not exhibit any learning deficit at any time point post-injury (sTBI/CEF/CYC 1 dpi: 17 ± 2.42 trials, *p* = 0.99, 14 dpi: 14 ± 1.59 trials, *p* = 0.99, Figure 6A). Similarly, preconditioning sTBI fish with purmorphamine blocked any significant learning deficit at any time (1–14 dpi) following injury relative to undamaged controls, (sTBI/PUR 1 dpi: 19 ± 1.56 trials, *p* = 0.99, 14 dpi: 16 ± 1.7 trials, *p* = 0.98, Figure 6A).

We next assessed the potential of purmorphamine preconditioning to ameliorate the deficits seen in memory following TBI [17,28]. To assess immediate and delayed recall, we again used the shuttle box assay [28]. Untreated sTBI fish displayed significant immediate memory deficit, with a decrease of −48.77% ± 3.36% (*p* < 0.01) in successful trials when retested 4 hpi, while undamaged fish exhibited a slight increase of 5% ± 1.62% in successful trials when retested 4 h following testing period 1 (Figure 6B). sTBI fish that were purmorphamine preconditioned also displayed significant immediate memory deficits (−14.66% ± 2.99%, *p* < 0.01); however, they significantly outperformed untreated sTBI fish (*p* < 0.01, Figure 6B). Neither group displayed significant delayed memory deficits suggesting that prophylactic Shh activation may dampen the cognitive learning and immediate memory deficits observed following sTBI.

## 4. Discussion

Smo agonists have been demonstrated to play intricate roles in regenerative therapies [37,55] and purmorphamine has been shown to provide neuroprotective effects and greatly influence recovery of cognitive performance in rodents following neurological insult and aged postoperative cognitive dysfunction [22,37,56]. We examined the regulation of TBI-induced excitotoxicity through the mechanistic link between Shh signaling and Eaat2a. We demonstrated that inhibiting the Shh response following TBI exacerbated PTS, while preconditioning with purmorphamine significantly reduced multiple facets of injury-induced pathologies, rapidly increased cognitive recovery, and in some instances attenuated the deficit altogether. To our knowledge, the use of purmorphamine in zebrafish following any mechanism of TBI has not been examined. Although several studies have demonstrated the inhibition of cell proliferation in the zebrafish brain following cyclopamine [57,58,59], we could not find studies illuminating the persistence of injury-induced pathologies or cognitive deficits associated with the application of cyclopamine.

The need to further examine TBI-induced PTS and identify potential therapeutics has become more and more imperative. Over the past decade, the number of ASMs developed has increased, however these therapeutics largely rely on a similar mechanism of action and drug-resistant epilepsy has increased among patients with PTS [10,11]. Traditional first line ASMs are GABA agonists, and rodent models revealed refractory PTS to these ASMs [60,61]. Cho et al. [62] recently examined Valproate (valproic acid, VPA) and other first-line ASMs (Carbamazepine, and Phenytoin) in their adult zebrafish TBI-PTS model [15] and observed no significant reduction in seizure activity and persistent cognitive deficits. We recognized the potential difference in mechanism in inducing TBI and tested both first and second-line ASMs in our TBI model. We similarly observed no improvement in TBI-induced seizures, edema, and cognitive deficits (data not shown). In contrast, our findings that blocking glutamate receptors or increasing expression of the glutamate transporter Eaat2a reduced TBI-induced PTS supported similar observations of EAAT2 reducing PTS in other models [63,64,65].

Modulation of Shh signaling has been considered a potential therapeutic for various ailments, though much of this work focused on Shh inhibition and use of Smo antagonists as potential cancer targets [66,67]. ERIVEDGE^®^, the only current FDA-approved Smo antagonist [68], made it through clinical evaluation and is approved for advanced basal cell carcinoma. The mechanistic consequences of Shh activation are less known and long-term Shh activation may stimulate tumor growth [69]. Furthermore, the results of Shh signaling activators have been inconsistent. For example, in epileptic rodents, Feng et al. [70] reported that increased Shh expression exacerbated seizure activity and negatively regulated extracellular glutamate, while others reported data suggesting that Shh activity upregulated EAAT2 expression, reduced astrocyte reactivity, and combated excitotoxicity [45,46,71,72]. Other studies reported that Shh activation is neuroprotective [23,37,38,39] and is effective in regenerative therapies [35,73,74]. However, much work needs to be done to provide insights into the mechanistic actions contributing these positive neurological outcomes. Although our study is far from exhaustive, it suggests that low and short doses of prophylactic purmorphamine may provide a neuroprotective effect against TBI-induced PTS. This therapeutic protocol may be amendable to targeted populations at higher risk of TBI and may provide protection beyond current safety measures, such as helmets.

## Data Availability

Data are contained within this article.
